# The trans-well coculture of human synovial mesenchymal stem cells with chondrocytes leads to self-organization, chondrogenic differentiation, and secretion of TGFβ

**DOI:** 10.1186/s13287-016-0322-3

**Published:** 2016-04-26

**Authors:** Eva Johanna Kubosch, Emanuel Heidt, Anke Bernstein, Katharina Böttiger, Hagen Schmal

**Affiliations:** Department of Orthopedics and Trauma Surgery, Albert-Ludwigs University Medical Center Freiburg, Freiburg, Germany; Department of Orthopaedics and Traumatology, Odense University Hospital, Sdr. Boulevard 29, 5000 Odense C, Denmark; Department of Clinical Research, University of Southern Denmark, Odense, Denmark

**Keywords:** Synovial mesenchymal stem cell, Coculture, Differentiation, Chondrocytes, Synovium, Chondrogenesis

## Abstract

**Background:**

Synovial mesenchymal stem cells (SMSC) possess a high chondrogenic differentiation potential, which possibly supports natural and surgically induced healing of cartilage lesions. We hypothesized enhanced chondrogenesis of SMSC caused by the vicinity of chondrocytes (CHDR).

**Methods:**

Human SMSC and CHDR interactions were investigated in an in-vitro trans-well monolayer coculture over a time period of up to 21 days. Protein expression was analyzed using histology, immunostaining, or enzyme-linked immunosorbent assay. Additionally, mRNA expression was assessed by quantitative PCR.

**Results:**

After 7 days, phase-contrast microscopy revealed cell aggregation of SMSC in coculture with CHDR. Afterwards, cells formed spheres and lost adherence. However, this phenomenon was not observed when culturing SMSC alone. Fluorescence labeling showed concurrent collagen type II expression. Addition of transforming growth factor beta (TGFβ) to the cocultures induced SMSC aggregation in less time and with higher intensity. Additionally, alcian blue staining demonstrated enhanced glycosaminoglycan expression around SMSC aggregates after 1 and 2 weeks. Although TGFβ mRNA was expressed in all SMSC, the protein was measured with constantly increasing levels over 21 days only in supernatants of the cocultures. Considering the enhanced mRNA levels following supplementation with TGFβ, a positive feedback mechanism can be supposed. In line with the development of a chondrogenic phenotype, aggrecan mRNA expression increased after 7 and 14 days in the cocultures with and without TGFβ. Coculture conditions also amplified collagen type II mRNA expression after 2 weeks without and already after 1 week with TGFβ. There was no difference in collagen type I and type X expression between SMSC alone and the coculture with CHDR. Expression of both collagens increased following addition of TGFβ. mRNA data correlated with the intensity of immunofluorescence staining.

**Conclusions:**

Paracrine effects of CHDR induce a chondrogenic phenotype in SMSC possibly mimicking joint homeostasis. Coculture approaches may lead to a better understanding of cellular interactions with potential implications for cartilage repair procedures.

## Background

Cartilage lesions have a limited capacity for repair and cause osteoarthritis (OA), so the search for treatment alternatives is ongoing. Many current approaches focus on the use of mesenchymal stem cells, which may play a significant role in both natural and surgically supported cartilage repair. Previously it has been described that natural cartilage repair can occur, especially in osteochondral defects; however, “the repair was mediated wholly by the proliferation and differentiation of mesenchymal cells of the marrow” [1]. More recently, a possible role for synovial mesenchymal stem cells (SMSC) was highlighted [2]. Injection of bone marrow-derived mesenchymal stem cells (BMSC) in osteoarthritic knees resulted in a long-term improvement of clinical outcome parameters [3], and SMSC were successfully used for arthroscopically assisted cartilage repair resulting in improved MRI features, histology, and clinical outcome [4]. Different studies suggested that SMSC have the best chondrogenic potential compared with mesenchymal stem cells derived from other tissue sources [5]. Adherence of SMSC to cartilage was mediated by hyaluronan [6], a possible mechanism for how these cells may be enriched in cartilage lesions. When SMSC migrate or are surgically placed at the site of a cartilage defect, they are in the direct vicinity of chondrocytes (CHDR) in their natural habitat. For this specific situation, coculture models are a powerful instrument to define and clarify cell–cell interactions. Until now the emphasis of SMSC/CHDR cocultures was to show effects in acute [7] or chronic [8] inflammation. Hereby, it could be demonstrated that SMSC were able to secret typical cartilage markers such as aggrecan and decisively influence the course of inflammation. Furthermore, pellet cocultures of mesenchymal stem cells, usually bone marrow derived, and CHDR resulted in formation of hyaline structured cartilage showing partially an even higher quality than the CHDR control group [9]. This phenomenon was independent of certain culture conditions and cell sources [10]. The disadvantage of this experimental approach is the missing possibility to differentiate between paracrine effects and cell–cell interactions. The hypothesis of the study was that CHDR are able to induce a chondrogenic differentiation of synovial stem cells. Since we presumed paracrine signals originating from CHDR causing this phenomenon, a coculture model was chosen where the cells were separated by a filter. This model does not allow direct cell contact and mimics the biological situation of cells collecting in a lesion with only marginal contact to the original cartilage layer. For evaluation, the markers of chondrogenesis aggrecan and collagen type II, the marker of dedifferentiation collagen type I, and the hypertrophy marker collagen type X were analyzed on RNA and protein levels. Emphasis of the study was the histological observation of cell organization, the time frame, and the influencing cytokines.

## Methods

Isolation, culture, and importance of SMSC in inflammation have been described previously [7]. The cells’ preparation protocols were approved by the Ethics Committee of the University of Freiburg as part of the “Tissue Bank for Research in the Field of Tissue Engineering” project (GTE-2002) and the biobank “Osteo” (AN-EK-FRBRG-135/14). Cells from the same donors were used when comparing different culture conditions.

### Isolation of SMSC

The cell preparation was described before [7]. Briefly, synovial tissue was gathered during knee operations with arthrotomy and arthroscopies (*n* = 4, male/female 2/2, average age 42.7 ± 15.0 years). The degree of OA was evaluated on X-ray images using Croft’s modification of the Kellgren and Lawrence score (KLS). Cells were used only from patients with healthy joints (KLS ≤ 2). The harvested tissue was kept in DMEM F-12 medium (Lonza BioWhittaker, Basel, Switzerland) at 4 °C. Within 2 h the tissue was cut into small pieces, washed, and transferred into DMEM F-12 medium with 10 % FCS (Biochrom, Berlin, Germany), 1 % penicillin/streptomycin (P/S) (Invitrogen, Karlsruhe, Germany), 0.5 % gentamycin (Biochrom), and 3 % collagenase P (Roche, Mannheim, Germany). The suspension was digested during the next 4 h on a shaking incubator (200 rpm) at 37 °C. Subsequently the released cells were centrifuged, washed, and seeded in expansion medium DMEM F-12 (10 % FCS, 1 % P/S, 0.5 % gentamycin). SMSC were seeded on coated T-flasks with a density of 2500–5000 cells/cm^2^ for expansion. The cells were frozen after reaching confluence. Thawed cells were grown and used when reaching a log phase of growth (passage 1). These cells were not further enriched and are also known as synovial fibroblasts or type B synoviocytes [11, 12]. Characterization of these cells was done by FACS showing that combined expression of the stem cell markers CD44, CD73, CD90, and CD105 was present in 76 %, but the combined expression of the negative markers CD11b, CD19, CD34, CD45, and HLA-DR reached only 6.9 ± 1.7 %. Osteogenic, adipogenic, and chondrogenic differentiation was possible using standard protocols [13].

### Isolation of CHDR

Cell preparation was described before [14]. Briefly, CHDR were gained from femoral heads during hip arthroplasty operations (*n* = 6, male/female 5/1, average age 79.2 ± 8.2 years). The degree of OA was evaluated on X-ray images using Croft’s modification of the KLS score. Cells from patients with advanced OA (≥ grade 3) were not used for experiments. Within 8 h after surgery, the cartilage was separated from the bone and cut into small pieces, washed, and transferred into DMEM F-12 10 % FCS, 1 % P/S, 0.5 % gentamycin, and 3 % collagenase CLS type II (Biochrom). Minced cartilaginous tissue was then enzymatically digested during the next 16 h on a shaking incubator at 37 °C with 200 rpm. Subsequently the released CHDR were centrifuged, washed, and seeded in expansion medium DMEM F-12 (Lonza BioWhittaker) (10 % FCS, 1 % P/S, 0.5 % gentamycin). Expansion of CHDR was performed by seeding them on coated T-flasks with a density of 2500–5000 cells/cm^2^. The cells were frozen after reaching confluence. Thawed cells were grown and used when reaching a log phase of growth (passage 1).

### Coculture conditions

SMSC in the bottom and CHDR on top were separated in a trans-well culture with 0.4 μm inserts. As a basal culture medium, DMEM F-12 medium supplemented with 10 % FCS, 1 % P/S, and 0.5 % gentamycin was used. Cell viability was >95 % before starting the experiment. Half-media changes were performed three times per week. The initial seeding density was 20,000 cells/cm^2^. Experiments were repeated at least three times with cells from different donors (one patient for one experimental trial). The cells were not pooled. The total time of coculture was 21 days maximum. There were three different groups: SMSC alone, SMSC with CHDR, and SMSC with CHDR supplemented with transforming growth factor beta (TGFβ)-3. A concentration of 10 ng/ml TGF-β3 (R&D Systems, Minneapolis, MN, USA) was added to the positive controls.

### TGFβ enzyme-linked immunosorbent assay

TGFβ levels in supernatants were analyzed by enzyme-linked immunosorbent assay (ELISA) (R&D; and BioSource Deutschland GmbH, Solingen, Germany) according to the manufacturers’ instructions. Briefly, this assay employs the quantitative sandwich enzyme immunoassay technique. The microplate was precoated with a specific monoclonal antibody. Supernatants were applied to the wells and, after washing, HRP-conjugated specific antibody was added to the wells. Following the next wash, color development was proportional to the protein concentration and calculated by comparison with a standard. A colorimetric method was applied to quantify the total protein amount in the lavage fluids.

### Histology

Cover slides were coated with poly-d-lysine (0.1 mg/ml) (Merck Millipore, Billerica, Massachusetts, USA) at 37 °C (5 % CO_2_) for 60 min, then washed and dried overnight. Afterwards, SMSC were grown on the slides in coculture in 24-well-plates (Corning Incorporated, Corning, NY, USA). For alcian blue staining, the cells were fixed and stained using the PAS-staining Kit (Merck Millipore, Billerica, Massachusetts, USA). Briefly, staining with alcian blue was followed by incubation with periodic acid, Schiff reagent, and hematoxylin, whereupon each step was followed by washing. After mounting, histological pictures were analyzed using an Olympus BX51 microscope (Olympus Deutschland GmbH, Hamburg, Germany) with the software module Stream Motion adjusting only brightness and contrast.

### Immunohistology

Cells were fixed at –20 °C with methanol (Sigma-Aldrich, St. Louis, MO, USA) for 10 min; afterwards, they were washed with Dulbecco’s phosphate-buffered saline (DPBS; Gibco Invitrogen, Carlsbad, CA, USA). For blocking of unspecific binding sites, cells were incubated at room temperature with 5 % BSA (AppliChem GmbH, Darmstadt, Germany) in DPBS. Primary antibodies were diluted as follows: collagen type I (mouse monoclonalAb COL-1; abcam, Cambridge, UK) 1:500, collagen type II (rabbit polyclonalAb COL-2; abcam) 1:250, and collagen type X (mouse monoclonalAb COL-10; abcam) 1:750. After washing, the antibody working solutions were applied to the cells and incubated at 4 °C overnight in a humid chamber. After washing three times, the cells were incubated with the working solution of the secondary antibody: Alexa Fluor 488 goat anti-mouse IgG and Alexa Fluor 568 goat anti-rabbit IgG (Life Technologies, Grand Island, NY, USA) in 1 % BSA, dilution 1:250. After washing three times again, color reagent (ProLong® Gold antifade reagent DAPI; Life Technologies) was applied. An Olympus BX51 microscope (Olympus Deutschland GmbH) with special fluorescence filters was used for image acquisition. The program ImageJ (Wayne Rasband, NIH; imagej.nih.gov/ij/download) facilitated overlaying of images.

### Real-time PCR

Real-time PCR was carried out for SMSC only. RNA samples from days 7 and 14 were transcribed into cDNA; RNA analysis was carried out for gene expression of aggrecan, TGFβ, and collagen type I, II, and X. Total mRNA was prepared using the Qiagen RNeasy kit according to the manufacturer’s instructions (Qiagen, Hilden, Germany). Total RNA (1 μg) was treated with 1 U DNAse I (Invitrogen, Karlsruhe, Germany) to remove genomic DNA. Poly-T primed cDNA synthesis was performed using 1 U reverse transcriptase III (RTIII; Invitrogen) per 1 μg RNA according to the manufacturer’s instructions. TaqMan™ PCR assays were performed in 384-well plates in a Roche LightCycler480 (Roche) using the Roche LightCycler Mastermix. For gene expression analyses, Roche’s universal ProbeLibrary Probes and recommended Universal ProbeLibrary Reference Gene Assays were used. The cycling conditions were denaturation (one cycle: 50 °C for 120 sec, 95 °C for 600 sec), followed by 40 amplification cycles (95 °C for 15 sec, 60 °C for 60 sec, 72 °C for 10 sec), followed by melting (one cycle: 95 °C for 10 sec, 50 °C for 30 sec, 70 °C for 1 sec), and cooling (one cycle: 40 °C for 30 sec). Data were quantified via ΔΔCT comparisons. Data were normalized by comparing genes of interest versus reference genes (GAPDH). Reaction efficiency is controlled by a relative standard curve and/or a calibrator per reaction. Real-time PCR was carried out in quadruplicate, each value representing an average of four experiments.

### Data analysis and statistics

Concentrations of cytokines determined by the specific ELISA were calculated according to the manufacturers’ instructions (R&D; and Thermo Scientific, Rockford, IL, USA), creating a standard curve and reducing data with a four-parameter logistic (4-PL) curve fit using GraphPad Prism 5 software (GraphPad Software, Inc., La Jolla, CA, USA). All values were expressed as mean ± standard error of the mean. Statistical significance was tested nonparametrically using the Mann–Whitney *U* test. The values of different time points were compared in each group, and the values of one time point were compared between the groups. Statistical significance was defined as *p* < 0.05.

## Results

### Histology

**Fig. 1:**
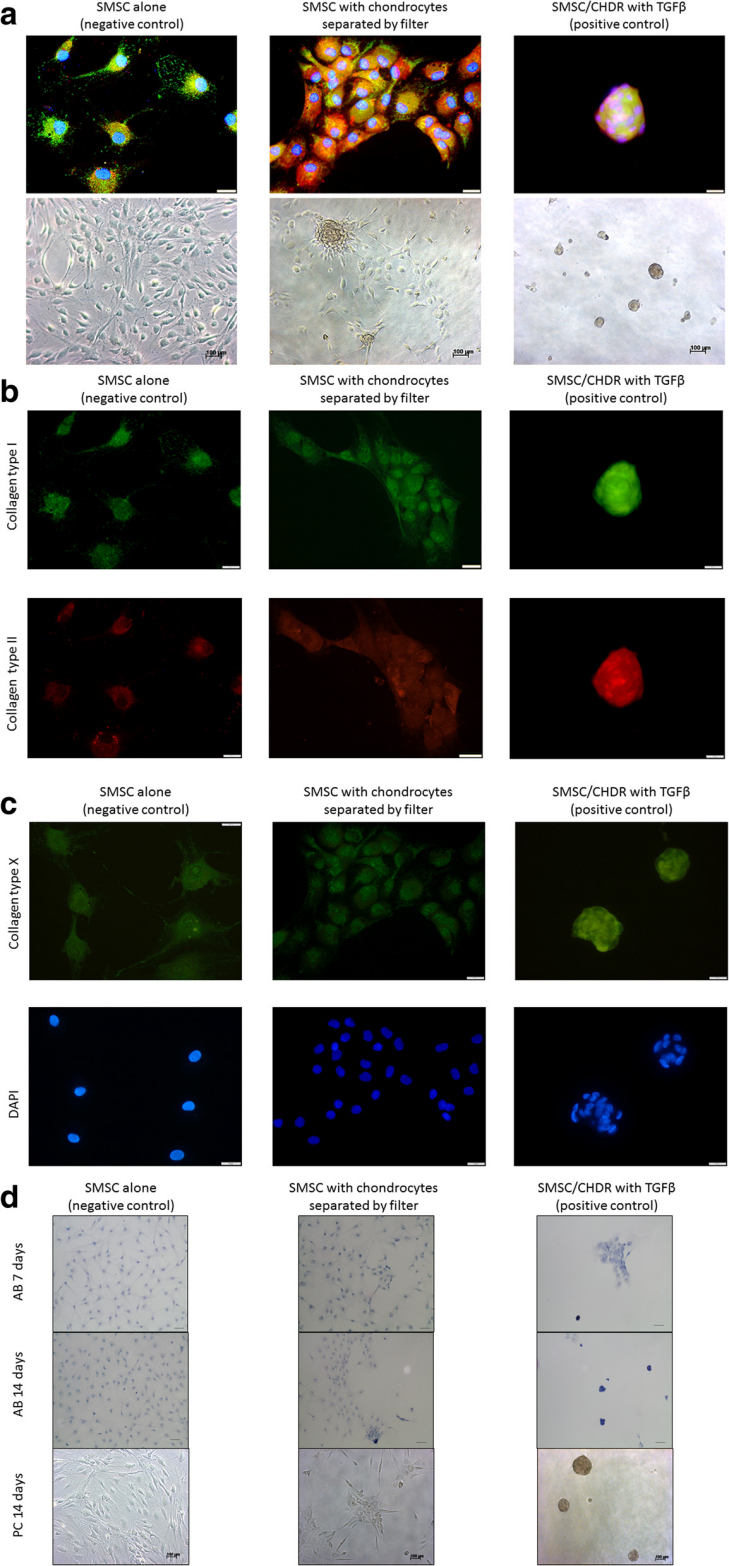
**a** Self-organization of SMSC after 7 days in monolayer culture. SMSC alone (*left*) stay separated, but in coculture with CHDR (*middle*) an aggregation of cells is visible, and addition of TGFβ (*right*) results in sphere formation. *Upper row* shows overlaying immunofluorescence staining (*green*: collagen type I, *red*: collagen type II, *blue*: DAPI; scale 20 μm), and *lower row* the phase-contrast microscopy. **b** Single staining for collagen type I (*upper row*) and type II (*lower row*) of the different groups. **c** Single staining for collagen type X and DAPI of the different groups. **d** Alcian blue (*AB*) staining of the different groups and time points. *Upper row*: AB 7 days (marker 50 μm), *middle row*: AB 14 days (marker 100 μm), *lower row*: phase-contrast (*PC*) microscopy 14 days (marker 100 μm). *CHDR* chondrocytes, *SMSC* synovial mesenchymal stem cells, *TGFβ* transforming growth factor beta (Color figure online)

SMSC were kept in monolayer cultures alone or in coculture with CHDR. As a positive control, these cocultures were supplemented with TGFβ. While SMSC alone stayed separated after 1 week, an aggregation of SMSC was visible in the coculture group. The addition of TGFβ even resulted in sphere formation (Fig. 1a–c). No further time points are shown, because the spheres lost adherence and could no longer be comparably stained. Since spin-downs resulted in cell debris, SMSC were grown on coated cover slides allowing only alcian blue staining and phase-contrast microscopy as shown in Fig. 1d. Again, the phenomenon of cell aggregation, sphere formation, and loss of adherence could be observed after 1 or 2 weeks, first in the TGFβ-treated positive control and then in the coculture. The alcian blue staining, which was more intense in cell aggregates, documents the presence of glycosaminoglycans/mucopolysaccharides. Cell aggregation could be observed with the cells of all different donors. Figure 1a compares the different groups by overlaying immunofluorescence staining and phase-contrast microscopy images. Figure 1b shows the single staining for collagen type I and type II of the different groups. The highest intensity but also the highest cell density was observed in the TGFβ-treated positive control group. Figure 1c demonstrates representative slides of the single staining for collagen type X and the DAPI staining for the different groups. The percentage-wise estimation of the aggregation extent was 0 % for SMSC alone, ≥40 % for the coculture of SMSC with CHDR, and ≥90 % for the coculture supplemented with TGFβ (overview in phase-contrast microscopy). Additionally, cell spheres per field of view were counted resulting in 0 ± 0 aggregates/field for SMSC alone, 1 ± 0.4 aggregates/field for the coculture of SMSC with CHDR, and 2.25 ± 0.5 aggregates/field for the coculture supplemented with TGFβ (*n* = 5, 20-fold magnification). There was no difference comparing the aggregation after 7 or 14 days.

### Role for TGFβ

**Fig. 2:**
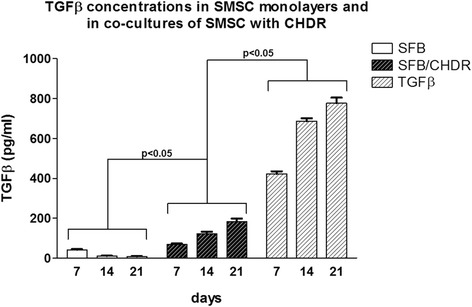
TGFβ concentrations in supernatants comparing SMSC in monolayer with the coculture of SMSC and CHDR without or with TGFβ supplementation. There is a statistically significant difference between the groups and each time point within the cocultures. *CHDR* chondrocytes, *SMSC* synovial mesenchymal stem cells or SFB synovial fibroblasts, *TGFβ* transforming growth factor beta

**Fig. 3:**
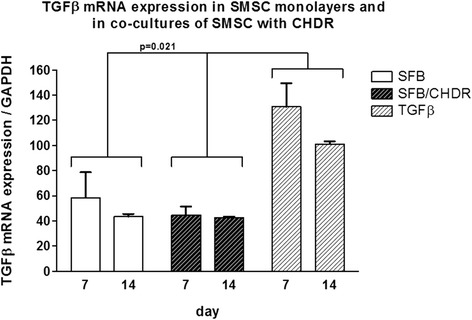
TGFβ mRNA expression comparing SMSC in monolayer with the coculture of SMSC and CHDR without or with TGFβ supplementation. There is a statistically significant difference between the TGFβ-supplemented group and both other groups, but not between the different time points within each group. *CHDR* chondrocytes, *SMSC* synovial mesenchymal stem cells or SFB synovial fibroblasts, *TGFβ* transforming growth factor beta

TGFβ concentrations in the supernatants were measured comparing SMSC in monolayer with the coculture of SMSC and CHDR without or with TGFβ supplementation (Fig. 2). As expected, the highest concentrations were observed in the positive control with TGFβ (777 ± 28 pg/ml). This is lower than the added concentrations indicating receptor immobilization of the cytokine or degradation, because supernatants were collected together with medium change. Concentration levels are followed by the coculture without TGFβ supplementation starting at week 1 with 68 ± 5 pg/ml and steadily increasing up to 183 ± 15 pg/ml at week 3. Although TGFβ was also found in the SMSC monolayer, levels were short over the detection limit. There was a statistically significant difference between the levels of each time point of all groups and each time point within the cocultures (*p* < 0.05). Because the statistical significance reached was marginal, additional comparisons were calculated using a Student’s *t* test, resulting in *p* < 0.02. Furthermore, the values of all groups were merged independent of the time point. The comparison resulted in highly significant differences (*p* < 0.001) using the direct comparison of groups with the Mann–Whitney *U* test and using the Kruskall–Wallis *H* test (multiple comparisons). Considering a significance level of the direct group comparisons very close to the defined α and a possibly not complete random sample, the tests may overstate the accuracy of the results. TGFβ mRNA expression was also compared over a 2-week interval (Fig. 3). A statistically significant difference was found between the TGFβ-supplemented group and both other groups (*p* = 0.021), but not between the different time points within each group.

### mRNA regulation of aggrecan and collagen type I, II, and X

**Fig. 4:**
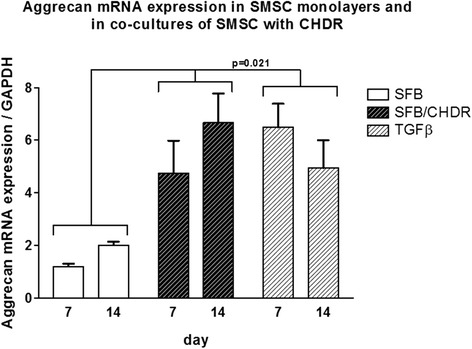
Aggrecan mRNA expression comparing SMSC in monolayer with the coculture of SMSC and CHDR without or with TGFβ supplementation. There is a statistically significant difference between both cocultures and the monolayer, but not between the different time points within each group. *CHDR* chondrocytes, *SMSC* synovial mesenchymal stem cells or SFB synovial fibroblasts, *TGFβ* transforming growth factor beta

**Fig. 5:**
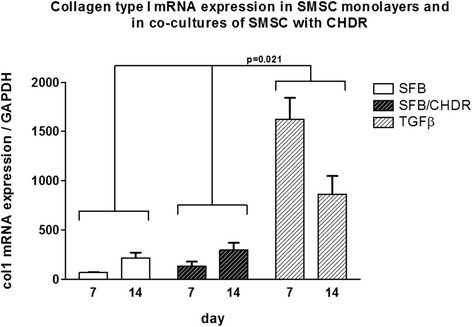
Collagen type I (col1) mRNA expression comparing SMSC in monolayer with the coculture of SMSC and CHDR without or with TGFβ supplementation. There is a statistically significant difference between the TGFβ-supplemented group and both other groups, but not between the different time points within each group. *CHDR* chondrocytes, *SMSC* synovial mesenchymal stem cells or SFB synovial fibroblasts, *TGFβ* transforming growth factor beta

**Fig. 6:**
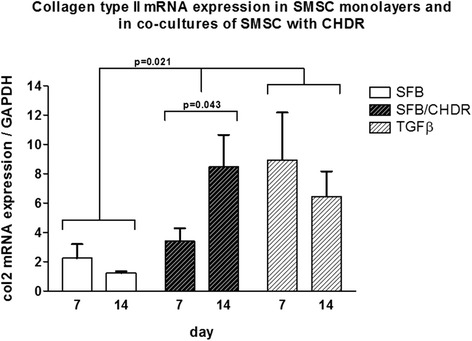
Collagen type II (col2) mRNA expression comparing SMSC in monolayer with the coculture of SMSC and CHDR without or with TGFβ supplementation. There is no statistically significant difference between the TGFβ-supplemented group and day 14 of the coculture, but these values are higher compared with SMSC alone and day 7 of the coculture. There is no difference between the different time points within each group except for the coculture. *CHDR* chondrocytes, *SMSC* synovial mesenchymal stem cells or SFB synovial fibroblasts, *TGFβ* transforming growth factor beta

**Fig. 7:**
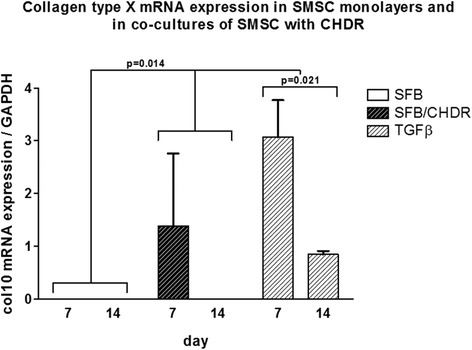
Collagen type X (col10) mRNA expression comparing SMSC in monolayer with the coculture of SMSC and CHDR without or with TGFβ supplementation. The value of the TGFβ-supplemented group at day 7 is statistically significantly higher than at all other time points. There is no difference between the different time points within the SMSC and the coculture groups. *CHDR* chondrocytes, *SMSC* synovial mesenchymal stem cells or SFB synovial fibroblasts, *TGFβ* transforming growth factor beta

Aggrecan mRNA expression was compared in SMSC monolayer with the coculture of SMSC and CHDR without or with TGFβ supplementation (Fig. 4). The highest levels were found in both cocultures at both investigated time points (without TGFβ: 4.7 ± 1.2-fold at week 1 and 6.7 ± 1.1-fold after 2 weeks, with TGFβ: 6.5 ± 0.9-fold at week 1 and 4.9 ± 1.1-fold at week 2). There is a statistically significant difference between both cocultures and the monolayer, but not between the different time points within each group. Collagen type I (col1) mRNA expression was also compared between SMSC monolayer with the coculture of SMSC and CHDR without or with supplemented TGFβ (Fig. 5). The highest values were measured in the TGFβ-supplemented coculture (up to 1625 ± 219-fold). There is a statistically significant difference between the TGFβ-supplemented group and both other groups (*p* = 0.021), but not between the different time points within each group or SMSC alone and the nonsupplemented coculture. Collagen type II (col2) mRNA expression was also examined (Fig. 6), showing the highest values in both cocultures (up to 8.9 ± 3.2-fold). There was no statistically significant difference between the TGFβ-supplemented group and day 14 of the coculture, but these values were higher compared with SMSC alone and day 7 of the coculture (*p* = 0.021). There is no difference between the two time points within each group except for the coculture without TGFβ. Here the col2 mRNA increased between the first week and the second week significantly (*p* = 0.043). Since collagen type II is considered the main marker for differentiated cartilage, the results indicate increasing chondrogenic differentiation induced by the presence of CHDR in coculture and the addition of TGFβ. Furthermore, the mRNA of the chondrogenic hypertrophy marker collagen type X (col10) was analyzed (Fig. 7). Although we found significant values at day 7 in the plain coculture, the highest values were measured when TGFβ was supplemented (up to 3.1 ± 0.7-fold). The value in the TGFβ-supplemented group at day 7 is statistically significantly higher than at all other time points (*p* = 0.014). There is no difference between the different time points within the SMSC (0 caused by rounding) and the coculture groups.

## Discussion

The main finding of the study is that human CHDR are able to induce a chondrogenic phenotype in human SMSC in a trans-well coculture. Although SMSC were kept in a monolayer, chondrogenesis leads to loss of adherence and formation of spheres. This was sufficiently regulated by paracrine factors; no direct cell–cell interactions were necessary. The degree of cell self-assembly and sphere formation and the collagen type II and aggrecan expression were associated with the levels of TGFβ found in the supernatants. Collagen type X, a marker of chondrocyte hypertrophy, was expressed in the coculture of SMSC and CHDR when TGFβ was additionally supplemented.

Cocultures of mesenchymal stem cells and CHDR can result in improved chondrogenic differentiation in pellets [9] and immunological interactions including anti-inflammatory regulations attributed to mesenchymal stem cells [15]. The possible high impact of coculture systems on cartilage tissue engineering is documented by the increasing number of described technical solutions [16] and experimental designs [17]. In contrast to previous studies, this investigation focused on an experimental trans-well design using human synovial stem cells. This set-up was inspired by the idea that—unlike the previously suggested predominant role of bone marrow-derived progenitor cells for cartilage regeneration [1]—synovial stem cells might be able to contribute to natural cartilage regeneration. This phenomenon has been described, but the cellular sources for repair are controversially discussed. The potential of CHDR themselves appears to be very limited, as previously published outgrowth experiments have shown [18]. Since synovial fibroblasts are located in the direct vicinity of cartilage and cartilage lesions and also exhibit stem cell characteristics [19] with the best chondrogenic potential of different mesenchymal stem sources [5], these cells are an interesting candidate for the cellular origin of natural and spontaneous cartilage regeneration. The natural environment of cells, especially stem cells, determines their histological and biochemical phenotype [20]. Therefore, we hypothesized that CHDR are able to secret paracrine signals inducing a chondrogenic differentiation of SMSC. This was confirmed by showing spontaneous formation of first cell aggregation and then cell sphere formation by SMSC in coculture with CHDR. This was accompanied by expression of cartilage markers as aggrecan and collagen type II on the protein and mRNA levels. The key role for TGFβ in the described and observed process of chondrogenic differentiation was not only demonstrated for BMSC [21], but also for SMSC [22]. TGFβ has been described to upregulate collagen type I mRNA in osteoblasts [23] or CHDR [24]. Furthermore, TGFβ seems to induce the cartilage hypertrophy marker collagen type X. This was confirmed by our results, but high levels of either collagen type I or type X were mainly found in the control group that was supplemented with additional TGFβ. In accordance with these data, the hypertrophic status of chondrogenically differentiated mesenchymal stem cells has previously been described to be associated with overexpression of osteogenic markers as collagen type I and alkaline phosphatase [25], explaining our positive immunostaining and the higher collagen type I mRNA expression in the TGFβ-supplemented group.

The fact that exogenous TGFβ supplementation upregulated collagen type I and type X in the SMSC without providing further enhancement of collagen type II or aggrecan expression is probably a dosage effect, assuming that the CHDR are promoting chondrogenesis in SMSC by releasing TGFβ; or it could suggest that CHDR are releasing other paracrine factors modulating the effect. This has to be considered also in the light of the only marginal statistical significance regarding the increased TGFβ concentrations in the supernatants of the coculture. However, the CHDR alone had a chondrogenic effect (at least in terms of gene expression and sphere formation), causing minimal fibrochondrogenesis (col1) or hypertrophy (col10).

The observed missing accordance of TGFβ mRNA and protein regulation in the supernatants can have different causes. First, TGFβ protein might also significantly be secreted by CHDR, which in the current experimental set-up cannot be measured separately. Secondly, protein formation in SMSC might undergo further regulatory processing and not only depend on RNA regulation. The higher levels of TGFβ mRNA in the supplemented coculture suggest a positive feedback regulation.

Chondrogenesis of SMSC was also demonstrated in a pellet coculture model using rabbit CHDR that overexpressed TGFβ after adenoviral transfection [26]. Based on the results of our study it may be concluded that gene transfer might not be necessary, because the coculture itself provides sufficient paracrine stimuli for chondrogenic differentiation of SMSC. Similarly, TGFβ induced the chondrogenesis of SMSC with high levels of collagen type II, aggrecan, and Sox 9, and low levels of dedifferentiation or hypertrophy markers in a coculture pellet model using nucleus pulposus cells in serum-free medium [27]. All of the data emphasize the key role for TGFβ in chondrogenic differentiation for both in-vitro cultures of mesenchymal stem cells of different origin [13] and in the natural articular environment. Although the role of TGFβ seems striking, there are a few limitations attributed to the presented experimental set-up. First, the measured TGFβ levels in the supernatants are a summary response of all cells, which makes it impossible to differentiate the true origin. This is the nature of a coculture, and therefore mRNA levels were determined in SMSC. Unfortunately, the data were not completely conclusive, because TGFβ mRNA could also be found in SMSC alone, indicating separate regulation pathways for protein and mRNA. Secondly, the simple presence of TGFβ does not allow drawing functional conclusions. This means we observed an association of TGFβ in the supernatant and chondrogenic differentiation of SMSC, but the biological or mechanistic relation is not necessarily evident. However, the stimulatory success of the coculture with CHDR on the chondrogenic differentiation of SMSC underlines the potential role of these cells in natural and artificially supported cartilage repair.

## Conclusions

The vicinity of CHDR in a trans-well coculture induces a chondrogenic phenotype in SMSC. This process is associated with increased TGFβ secretion and offers possible implications for cartilage repair. This effect may play a significant role in natural and surgically induced cartilage repair, especially in cell therapeutic approaches.

## Abbreviations

BMSC: bone marrow-derived mesenchymal stem cells
BSA: bovine serum albumin
CHDR: chondrocytes
col1: collagen type I
col10: collagen type X
col2: collagen type II
DMEM: Dulbecco’s modified Eagle’s medium
DPBS: Dulbecco’s phosphate-buffered saline
ELISA: enzyme-linked immunosorbent assay
FCS: fetal calf serum
KLS: Kellgren and Lawrence score
OA: osteoarthritis
P/S: penicillin/streptomycin
PAS-staining: Periodic acid–Schiff stain
SMSC: synovial mesenchymal stem cells
TGFβ: transforming growth factor beta
